# Management Strategies for Isolated Orbital Floor Fractures: A Systematic Review of Clinical Outcomes and Surgical Approaches

**DOI:** 10.3390/diagnostics15233024

**Published:** 2025-11-27

**Authors:** Bayad Miran, Daniel J. Toneatti, Benoît Schaller, Ioanna Kalaitsidou

**Affiliations:** 1 Department of Cranio-Maxillofacial Surgery, Inselspital, Bern University Hospital, University of Bern, 3010 Bern, Switzerland; 2 Department of Biomedical Research Bern (DBMR), University of Bern, 3008 Bern, Switzerland; 3 Graduate School for Cellular and Biomedical Sciences (GCB), University of Bern, 3012 Bern, Switzerland

**Keywords:** orbit, orbital reconstruction, maxillofacial injuries, surgical procedures, operative, biocompatible materials, systematic review

## Abstract

**Background/Objectives:** Isolated orbital floor fractures are a common consequence of midfacial trauma and are frequently associated with functional and aesthetic complications such as diplopia, enophthalmos, infraorbital hypoesthesia, restricted ocular motility, and, in rare cases, blindness. Current therapeutic approaches vary significantly between different surgeons. This systematic review aimed to evaluate different treatment strategies for isolated orbital floor fractures to determine the most effective approaches. **Methods:** Electronic systematic searches were conducted using keywords to identify studies reporting isolated orbital floor fractures. Publications were screened for eligibility, and relevant data were extracted and evaluated. **Results:** This systematic review included 444 patients from 27 selected studies. 42 patients were treated conservatively, while 381 underwent various surgical interventions. Overall, the mentioned treatment modalities were successful in correcting enophthalmos (85.2%), diplopia (74.8%), ocular motility restriction (61.6%), and sensory disturbances (61.1%) in their respective patient cohorts. The complication and reoperation rates for the surgical interventions were low during the follow-up periods ranging from 6 weeks to 10 years. The timing, surgical approach, and reconstructive technique varied widely across the studies. **Conclusions:** Both conservative and surgical management of isolated orbital floor fractures can achieve satisfactory clinical outcomes. Clinical symptoms, defect size, and the surgeon’s preference define the ideal treatment modality.

## 1. Introduction

The orbital floor is a thin and fragile bone, composed of portions of the maxillary, zygomatic, and palatine bones, and is frequently fractured following trauma to the midface. Due to its delicate structure and anatomical location, it is especially prone to fracture following blunt facial trauma. These fractures may occur as “isolated” or “pure”, or in combination with other injuries, most commonly involving the zygomaticomaxillary complex.

Isolated orbital floor fractures represent approximately 22% to 47% of all orbital fractures [1,2]. They do not involve the orbital rim and are typically located medial to the infraorbital nerve canal or extend on both sides of the nerve [3]. The primary etiologies include assault, motor vehicle accidents (MVAs), sport-related trauma, and accidental falls to the midface [4,5,6].

Orbital floor fractures can lead to significant morbidity, with affected patients presenting with symptoms such as diplopia, enophthalmos, persistent hypoesthesia of the infraorbital nerve, and restricted ocular motility. In severe cases, these injuries may result in permanent visual impairment or even blindness [7,8,9,10,11,12,13,14].

Surgical intervention, when indicated, typically involves spanning the orbital floor defect with a material that provides structural support and restores orbital volume. The primary objective of surgical treatment is to effectively address anatomical and functional deficits. Common indications for surgical reconstruction include clinical evidence of entrapment, persistent diplopia, reflex bradycardia, clinical enophthalmos exceeding 2 mm, and fractures involving a substantial portion of the orbital floor [15,16,17,18]. Multiple studies have also confirmed that asymptomatic orbital fractures can be managed conservatively with favorable outcomes, provided there are no adverse radiological signs, such as entrapment or significant defects [17,19,20,21,22].

Despite decades of clinical experience, there remains a lack of consensus on optimal timing, choice of surgical approach, and selection of implant materials. Perioperative management protocols vary substantially across institutions. This systematic review aims to critically evaluate current conservative and surgical treatment strategies for isolated orbital floor fractures. Specifically, it assesses clinical outcomes, surgical techniques, antibiotic usage, and follow-up practices. These management decisions may influence patient outcomes, making an overview of the currently available literature essential for future treatment recommendations.

## 2. Materials and Methods

This systematic review was conducted in accordance with the PRISMA 2020 Statement, an updated guideline for the reporting of the study methodology [23]. Comprehensive electronic searches (until 17 September 2024) were performed across the Cochrane Central Register of Controlled Trials, Cochrane Database of Systematic Reviews, PubMed, EMBASE, and MEDLINE databases. Grey literature platforms were not included in the search process. The search strategy utilized keywords such as “treatment” or “management” in conjunction with “isolated orbital floor fractures”. No filters were applied during the electronic search. The results of the literature search were systematically imported into a Microsoft Excel file (Microsoft Excel 2021, Microsoft Corporation, Redmond, WA, USA) for organizational and analytical purposes. First, duplicate entries were identified and removed. Eligibility screening was initially performed based on the study title and abstract. In the second screening phase, full-text articles of the remaining studies were reviewed in detail. The references of all included studies were examined to identify additional literature. Only studies investigating isolated orbital floor fractures and providing precise clinical diagnostic and outcome data were included. Articles with a different focus or which did not report clinical, diagnostic or outcome data were excluded. We also excluded studies addressing comminuted orbital floor fractures or fractures of the zygomaticomaxillary complex. Additional articles were excluded if they included patients younger than 18 years and lacked individual data to exclude these patients. Furthermore, case reports, articles published in languages other than English, French, or German, and studies without available full-text versions were excluded. All relevant data were extracted from the included studies. These data encompassed the study design, the number of patients, patient demographics (age and sex), year of observation, average duration of follow-up, type of treatment (conservative vs. surgical), mean time to intervention, surgical approach, and method of reconstruction. The mean surgical intervention duration was determined, and details regarding pharmacological treatments, complications, and required postoperative computed tomography (CT) scans were documented. The various causes of orbital floor fractures were systematically recorded. The complete clinical preoperative and postoperative ophthalmic examinations, including evaluations of enophthalmos, diplopia, sensory disturbances of the infraorbital nerve, and ocular motility, were documented in detail. Relevant data were systematically extracted, summarized in tabular form, compared, and subsequently analyzed.

The methodological quality of all the included studies was assessed using the Joanna Briggs Institute (JBI) Critical Appraisal Checklist for Cohort Studies [24].

These steps were conducted twice independently by a single examiner to increase reliability. Uncertainties were discussed with the co-investigators until agreement was reached.

## 3. Results

**Figure 1:**
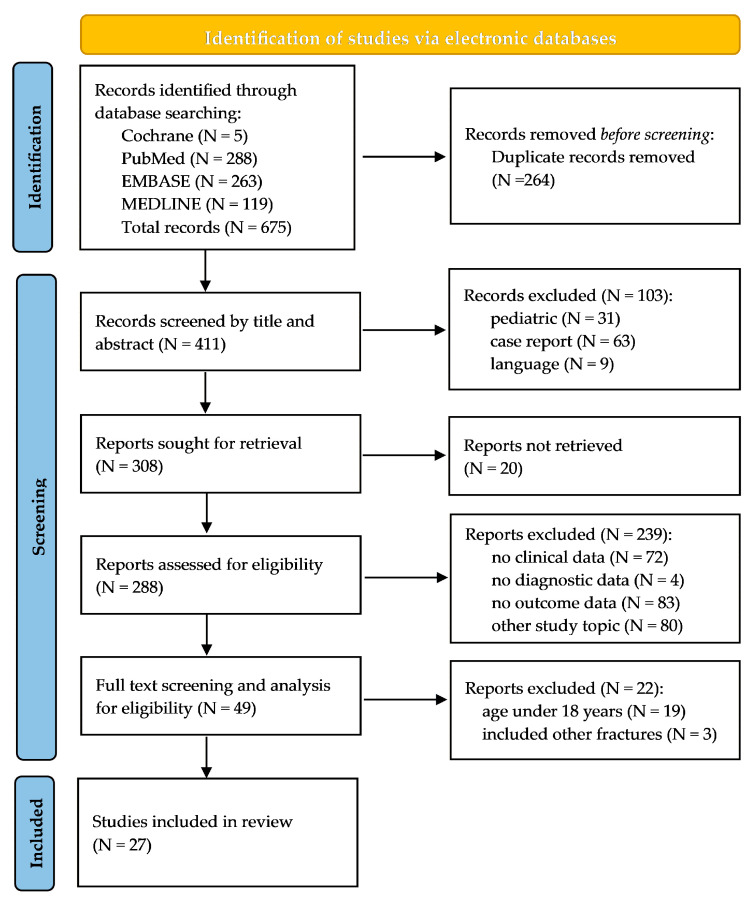
Flowchart illustrating the study selection process in accordance with the PRISMA (Preferred Reporting Items for Systematic Reviews and Meta-Analyses) guidelines [23].

The electronic literature search for this systematic review yielded 675 articles (Figure 1). After removing duplicates, 411 studies underwent title and abstract screening: During this process, 103 studies were excluded for irrelevance to the research question. An additional 20 studies were inaccessible in full text, leaving 388 studies for full-text screening. Of these, 239 studies did not meet the inclusion criteria. Subsequently, 49 studies proceeded to full-text screening and eligibility analysis. At this stage, 22 studies were excluded: 19 due to inability to isolate and exclude cases involving patients under 18 years of age, and 3 for including other orbital wall fractures. Ultimately, 27 studies met all eligibility criteria and were included in this systematic review (Figure 1).

A detailed summary of the included studies and their extracted data is presented in Table 1 [7,25,26,27,28,29,30,31,32,33,34,35,36,37,38,39,40,41,42,43,44,45,46,47,48,49,50].

The systematic review incorporates 23 retrospective and 4 prospective studies. Overall, 444 patients, comprising 72% males and 28% females, were included. The mean age of the patients was 36 years (range: 18–91 years). Observation took place between 1992 and 2020, with an average follow-up duration ranging from 6 weeks to 120 months. Of the total cohort, 42 patients were treated conservatively, while 381 underwent surgical intervention.

Of the patients requiring surgical intervention, 112 were treated within 1 week, 244 within 1 to 2 weeks, and 12 within 2 to 3 weeks after trauma. Furthermore, in 11 cases, treatment was carried out after 3 weeks. Notably, Scawn et al. (2016) reported 10 cases of delayed surgical intervention, ranging in age from 7 weeks to 21 years [30]. Cheong et al. (2010) documented 1 case in which surgical intervention was performed 5 years post-injury [50].

A variety of surgical approaches were reported across the included studies. Thirteen studies (218 patients) exclusively used external approach techniques. Of those, 6 studies (82 cases) used transconjunctival incisions; in 25 cases, only subciliary approaches were utilized; 4 cases were performed via pre-existing lacerations, 28 cases employed mid-eyelid approaches, and 2 cases utilized a mediopalpebral access route. Gugliotta et al. (2023) reported 18 cases managed using transconjunctival or subciliary incisions [31]. Ethunandan and Evans (2011) reported 3 cases using transcutaneous or transconjunctival incisions [45]. Al-Qattan and Al-Qattan (2021) reported 7 cases managed via only infraorbital approach [46]. The repair of the orbital floor using exclusively transmaxillary endoscopy was reported in 8 studies, encompassing a total of 111 cases. In 3 studies, 18 cases were described, in which the orbital floor was repaired exclusively through endoscopic endonasal access using an antral balloon catheter [27,37,44]. Additionally, a combination of external and endoscopic approaches was documented in 4 studies, involving 34 patients. Notably, Polligkeit et al. (2013) reported a combined transmaxillary and subciliary approach for the reconstruction of the orbital floor in 13 patients [40].

Various reconstructive materials were employed for orbital floor repair. Autologous grafts were utilized in 5 studies, involving the repair of 20 orbital floors using autogenous iliac crest bone [34], 2 cases with unspecified bone grafts [25], and 9 cases using grafts from the maxillary wall in the study by Emodi et al. (2018) [49]. A wide range of alloplastic materials were used and documented for orbital floor reconstruction. These included over 23 cases with Medpor^®^ implants (Stryker, Kalamazoo, MI, USA) [29,30,44,50], 2 cases with Nylon Foil (SupraFOIL^®^ Smooth Nylon Foil, Supramid, S Jackson Inc., Alexandria, VA, USA) [30], 12 cases with Ethisorb^®^ patches (Ethicon, Norderstedt, Germany) [37,38,39,40], 7 cases with PDS™ sheets (Ethicon™ PDS™ Sheet ZX-8; Ethicon Inc., a subsidiary of Johnson & Johnson, Somerville, NJ, USA) [40], 5 cases with LactoSorb^®^ (PLLA/PGA implant, W. Lorenz Surgical, Inc., Jacksonville, FL, USA) [43], and 3 cases with MacroPore^®^ implant (MacroPore Biosurgery Inc., San Diego, CA, USA) [45]. Gugliotta et al. (2023) presented 18 cases where Lyoplant^®^ (Braun, Tuttlingen, Germany), a collagen-based, biologically derived membrane implant, was used for orbital floor reconstruction [31]. Antral balloon catheters were reported in 4 studies (endoscopic endonasal or transmaxillary approach), with their use documented in 73 cases [27,37,39,44]. Titanium mesh implants were used in 42 cases across 7 studies, while preformed standardized titanium mesh plates were used in 37 cases documented in five studies.

The mean duration of surgical interventions is summarized in Table 2. The duration of transmaxillary endoscopic orbital floor surgery is reported to range from 60 to 120 min [7,29]. In the comparative study by Reich et al. (2014) [33], the reconstructions using polydioxanone (PDS) sheets averaged 79 min, while the application of transmaxillary ballon catheter techniques took 82 min. Procedures involving preformed titanium mesh plates were considerably longer, with a mean duration of 110 min [33].

Sigron et al. (2020) [48] compared freehand bent titanium mesh implants with pre-bent patient-specific titanium mesh implants. The mean duration was 99.8 ± 28.9 min using freehand bent implants versus 57.3 ± 23.4 min in the pre-bent group [48].

The underlying causes of 268 isolated orbital floor fractures are detailed in Table 3. The most common cause was assault, accounting for 94 cases (35.1%), followed by falls (58 cases, 21.6%), sports accidents (51 cases, 19%), and motor vehicle accidents (MVAs, 37 cases, 13.8%). Less frequent reasons included workplace injuries (3 cases, 1.1%) and gunshot wounds (1 patient, 0.4%). Three cases (1.1%) were simply attributed to miscellaneous causes.

In Table 4, preoperative clinical findings of enophthalmos greater than 2 mm were compared with postoperative outcomes. Across the included studies, preoperative enophthalmos was present in 88 of 262 patients (33.6%). Following surgical intervention, only 13 patients (5.0%) still exhibited enophthalmos greater than 2 mm, demonstrating a substantial overall improvement. Among those with preoperative enophthalmos, 75 patients (85.2%) achieved complete resolution, whereas enophthalmos persisted in 13 patients (14.8%). When examining the individual studies, differences in postoperative outcomes became apparent. For example, most reports, including Nahlieli et al. (2007), Scolozzi et al. (2009), and Scawn et al. (2016), described complete resolution of preoperative enophthalmos with no residual cases [7,26,30]. In contrast, O’Connell et al. (2015), Soejima et al. (2013) and Jin et al. (2007) documented a small number of patients with persistent or newly developed enophthalmos [34,39,44]. Sigron et al. (2021) reported one case of new postoperative exophthalmos, attributed to swelling, highlighting that new orbital volume changes may occur as a transient complication [38]. Most cases (7 of 13) of postoperative enophthalmos reported in this systematic review were described by Prabhu et al. (2021) without detailed case descriptions [28].

The comparison of preoperative and postoperative clinical diplopia outcomes is presented in Table 5. Of the 398 patients analyzed, 262 (65.8%) reported preoperative diplopia in certain fields of gaze. Postoperatively, residual diplopia was observed in 66 patients (16.6%). Among the 262 patients with diplopia prior to treatment, 196 (74.8%) achieved complete resolution, while 66 cases (25.2%) showed persistence.

Across the included studies, many reports, such as those by Nahlieli et al. (2007), Yano et al. (2010), and Fernandes et al. (2007), stated that preoperative diplopia resolved completely after treatment [7,25,29]. Others, for instance, Reich et al. (2014), O’Connell et al. (2015), and Polligkeit et al. (2013), described postoperative diplopia only during extreme movements, with little or no impact on daily functioning [33,34,40]. More pronounced cases were also noted. Scolozzi et al. (2009) described significant postoperative upward diplopia caused by vertical motility restriction, which resolved after implant removal [26]. Scawn et al. (2016) reported a case of persistent diplopia linked to inferior rectus atrophy or scarring, despite correct implant positioning [30]. Similarly, Sigron et al. (2021) observed both unresolved diplopia and newly developed diplopia following surgery, again mostly during extreme gaze [38]. Prabhu et al. (2021) reported multiple cases of persistent or unclear diplopia but did not provide detailed case data) [28].

Table 6 compares the preoperative and postoperative clinical outcomes associated with sensory disorders of the infraorbital nerve. Among 78 patients, 36 (46.2%) reported preoperative hypesthesia, while 14 patients (17.9%) continued to experience these symptoms postoperatively. Thus, 61.1% of patients with preoperative hypesthesia achieved resolution, whereas 38.9% had persistent symptoms.

In several reports, such as those by Nahlieli et al. (2007) and Scawn et al. (2016), most cases of preoperative hypesthesia improved or resolved, though isolated cases of new, temporary hypesthesia after surgery were also observed [7,30]. Yano et al. (2010) described one such transient case, which resolved without intervention [25]. Both Homer et al. (2019) [41] and Sigron et al. (2021) [38] compared sensory disturbances in surgical and conservative management. They showed sensory recovery in the surgically treated group in 2 of 7 and 7 of 14 patients, respectively, while in the conservatively treated groups, only 1 of 3 and 0 of 4 patients recovered fully [38,41].

The comparison of preoperative and postoperative findings related to ocular motility are presented in Table 7. Ocular motility restriction was documented preoperative in 86 (55.1%) of the 156 patients. Postoperatively, 32 patients (37.2%) still exhibited ocular motility restriction. Surgery was successful in improving ocular motility in 54 (62.8%) cases. A patient with persistent, significant diplopia on downgaze, as reported by Scawn et al. (2016), experienced worsened ocular motility postoperatively [30]. Sigron et al. (2021) documented three cases of persistent but mild ocular motility impairment and one case that did not improve [38]. Polligkeit et al. (2013) mention improvement in all cases after surgery, with 3 cases of slight and 1 case of clear restriction persistence [40].

Across the included studies, perioperative antibiotic prophylaxis was routinely administered, most commonly with amoxicillin–clavulanic acid, although the exact dosage, route, and duration varied considerably [7,26,37,40,42,50]. Several studies also incorporated corticosteroids, either intravenously or orally, yet detailed protocols were lacking in most cases [7,27,30,31]. Supportive measures, such as nasal decongestants, soft-diet recommendations, and oral hygiene care, were inconsistently described and, in most studies, were not reported or implemented [40,42,50].

Similarly, postoperative radiological assessment was highly variable between the studies. Nahlieli et al. (2007) and Fernandes et al. (2007) (15 cases) used postoperative CT scans within 24 h to confirm correct implant placement and assess the orbital floor, overall orbital condition, and maxillary sinus [7,29]. Reich et al. (2014) relied on conventional cranial eccentric skull X-rays for five uncomplicated cases, while CT imaging was conducted in another five [33]. Abdelazem et al. (2020) scheduled follow-up appointments three months post-surgery, including CT scan evaluations [42]. In contrast, Ikeda et al. (1999) did not routinely perform postoperative computed tomography or magnetic resonance imaging [27].

Among the 568 surgical cases included, the reoperation rate was low. Prabhu et al. (2021) reported that six patients (8.3%) required reoperation without providing further details [28]. Polligkeit et al. (2013) described three cases in which revision surgery was necessary due to an inadequate initial repair performed solely via a subciliary approach [40]. O’Connell et al. (2015) also reported one case that required immediate re-exploration in the recovery room due to ocular pain and swelling, although no hematoma or active bleeding was found during re-exploration [34].

A summary of the critical appraisal evaluation can be found in Appendix A.

## 4. Discussion

The clinical outcomes of the cases included in this systematic review, mainly related to surgical interventions, showed consistently good aesthetic and functional results, with a low complication and reoperation rate. Enophthalmos and diplopia resolved in 85.2% (75 of 88) and 74.8% (196 of 262), respectively, while ocular motility restriction and infraorbital nerve dysfunction returned to normal in 61.6% (53 of 86) and 61.1% (22 of 36) of cases. Despite considerable heterogeneity in surgical approaches, the timing of intervention, and postoperative protocols, most patients achieved sufficient restoration of orbital anatomy to regain function.

With respect to etiology, interpersonal violence was the most frequent cause of isolated orbital floor fractures (35.1%). The exact distribution, however, varies depending on geographic, cultural, and socioeconomic factors [51,52,53,54]. Several included studies did not specify fracture etiology, limiting a comprehensive assessment of regional differences.

Diagnostic evaluation focuses primarily on clinical examination and computed tomography (CT) imaging, which reliably assesses fracture size and soft-tissue entrapment and guides the decision between conservative and surgical treatment. Additional imaging modalities such as magnetic resonance imaging (MRI) were only selectively used and did not substantially influence management in the reviewed studies.

When considering surgical indications and timing, conservative management remains appropriate for non-displaced fractures without functional or cosmetic impairment. Standard protocols include a two-week observation period with analgesia, ocular mobility exercises, and close clinical follow-up [8].

Surgical intervention is primarily guided by functional impairment. Persistent diplopia, radiological entrapment, or clinically relevant enophthalmos remain the key indications for surgery, whereas reported size thresholds vary considerably among studies. As outcomes did not differ across different defect-size criteria in the reviewed literature, clinical symptoms appear more decisive than absolute measurements in determining the need for operative treatment [8,29,38].

The optimal timing of surgery remains a matter of debate. The available evidence suggests that immediate repair (<48 h) is required in trapdoor fractures or in the presence of a clinically relevant oculocardiac reflex [31,46,55,56,57]. In patients presenting with persistent diplopia or radiological entrapment, most studies recommend intervention within 14 days [30,58,59]. In the absence of these findings, a short delay beyond two weeks may be acceptable, as several studies did not demonstrate inferior outcomes with later repair [60].

Concerning surgical approach, various techniques are available, each suitable for different fracture patterns. Endoscopic techniques, including the endoscopic endonasal and endoscopic transmaxillary approaches, have been associated with reduced soft-tissue trauma, less infraorbital nerve hypesthesia, and shorter hospital stay [27], although their use is limited in lateral fractures, and some studies report sensory disturbances for the endoscopic transmaxillary approach [39]. External approaches remain widely used due to their reliable exposure and direct visualization. The transconjunctival incision shows consistently low complication rates and favorable cosmetic results, whereas the subciliary incision carries a higher risk of ectropion [61,62,63]. Worthington additionally emphasized the subtarsal or subconjunctival incision with lateral canthotomy as providing sufficient access and good aesthetic outcomes [32]. Combined approaches, such as a combined endoscopic transmaxillary–external approach, may be advantageous in extensive fractures or persistent diplopia, offering improved visualization of posterior defects and facilitating accurate reduction [40,64,65]. Our data show that clinical success can be achieved with different surgical techniques. A direct comparative evaluation of the different surgical approaches was not possible, as most included studies did not provide sufficient individual-level data to allow meaningful subgroup analyses. Therefore, individual assessment of the indicated technique is required.

The choice of reconstructive material depends largely on defect size and fracture complexity. Autogenous bone grafts remain a reliable option with good biocompatibility, but their use is limited by donor-site morbidity and variable resorption [49,66,67,68,69]. Alloplastic implants are therefore widely used. Resorbable materials, such as polylactic acid meshes, are suitable for small defects due to limited long-term stability [27,70,71], whereas non-resorbable implants, especially titanium meshes and porous polyethylene, are preferred for larger or complex fractures, providing stable long-term support [11,46,72,73,74,75,76,77,78,79,80,81]. Titanium meshes continue to represent the standard for extensive defects, and recent developments in pre-bent and patient-specific implants have improved anatomical accuracy and operative efficiency, although their higher cost remains a limitation [26,38,48,82,83,84,85,86,87,88,89,90,91,92,93,94,95,96,97].

Perioperative regimens varied considerably across studies, particularly regarding antibiotic and corticosteroid use. No direct comparisons were available, but infectious complications were rare regardless of protocol [26,30,31,33,37,40,42,50]. The low infection rate suggests that prolonged antibiotic courses are generally not necessary.

Postoperative CT imaging is not routinely required in uncomplicated courses, as intraoperative forced duction testing and unrestricted ocular motility are usually sufficient indicators of adequate reconstruction [32]. Imaging should be reserved for unclear symptoms, complex fractures, or suspected complications.

Follow-up periods ranged between 6 and 12 months in most studies, with prolonged monitoring recommended in cases of persistent diplopia or sensory disturbances. Diplopia typically improves within the first months, although nerve recovery may require considerably longer [26,86,98,99]. Regular reassessment helps to identify late complications such as enophthalmos or infection.

### Limitations and Outlook

This systematic review has several limitations. The study selection and data extraction were done by a single examiner. To mitigate bias, these processes were conducted twice independently. In cases of doubt, the studies were discussed with the co-investigators until agreement was reached. Further risk factors for selection bias include exclusion based on language and the omission of gray literature platforms. The relatively short average follow-up duration of 6 weeks in the included studies increases the risk of missing potential late-onset complications.

Although four studies reported on conservative management, evaluation of non-surgical approaches was limited by incomplete reporting [35,36,37,41].

The risk of bias analysis revealed that the studies included were of low overall quality. Most studies were of a retrospective nature, lacked a control group, and failed to report certain patient information and outcome measures. Furthermore, a clear distinction between the different surgical and conservative treatment groups was not consistently possible, leading to group heterogeneity, limited subgroup analyses, and reduced interpretability of treatment outcomes. This is especially important, as fracture characteristics (such as soft-tissue herniation or bone defect size) influence the surgical approach and the applied biomaterial, and are relevant for future complications [35].

All these factors influenced our decision not to conduct a meta-analysis and recommend careful interpretation of the collected data.

To achieve a more robust evaluation of treatment options for isolated orbital floor fractures, future research should prioritize prospective comparative studies with sufficient sample sizes and long-term observations. They should report on clinical examinations, imaging diagnostics, and assessments of fracture size (radiological and clinically at surgery) and analyze the chosen surgical approach, the applied biomaterial, and peri- and postoperative regimen based on standardized outcome metrics.

## Figures and Tables

**Table 1:** Overview of the selected studies, including study design and patient characteristics.

Source	Study Design	Patients [No.]	Patient Age, Mean and Range [y]	Sex, Male/Female [No.]	Year of Observation	Average/Range of Follow-Up Duration	Treatment Conservatively/Surgical [No.]	Average Time to Intervention	Surgical Approach	Method of Reconstruction (No. of Cases)
Nahlieli et al. (2007) [7]	retrospective	5	36.6, 24–47	5/0	NR	2–12 m	0/5	2 w	endoscopic transmaxillary	Titanium mesh implant (Synthes, Oberdorf, Switzerland)
Yano et al. (2010) [25]	retrospective	2	24.3, 18.8	2/0	2002–2007	NR	0/2	9 d	subciliary	Bone graft
Scolozzi et al. (2009) [26]	prospective	7	41.5, 21–71	5/2	05.2007–01.2008	6 w–9 m	0/7	NR	transconjunctival	Titanium mesh (MatrixORBITAL, Synthes, Switzerland)
Ikeda et al. (1999) [27]	retrospective	6	31.5, 26–38	5/1	09.1994–06.1997	6 m	0/6	2 w (2) 3 w (4)	endoscopic endonasal	Balloon catheter
Prabhu et al. (2021) [28]	retrospective	75	37.9	NR	2012–2019	20 w	0/75	3–14 d	Transconjunctival (49), transcutaneous (23), prior laceration (3)	NR
Fernandes et al. (2007) [29]	prospective	10	37.3, 19–47	7/3	06.2005–12.2005	12.7 w 1–26 w	0/10	10.9 d, 3–36 d	endoscopic transmaxillary	Medpor^®^ implant
Scawn et al. (2016) [30]	retrospective	10	55.1, 25–80	NR	04.2008–01.2014	8 m, 6 w–56 m	0/10	7 w–21 y	transconjunctival	Medpor^®^ implant (8), Nylon Foil (2)
Gugliotta et al. (2023) [31]	retrospective	18	25.3, 18–47	12/6	01.2006–12.2020	6 m	0/18	3.8 d, 0–17	transconjunctival, subciliary	Lyoplant^®^
Worthington (2010) [32]	retrospective	5	21.2, 19–23	5/0	1997–2007	3.7 m, 2–6 m	0/5	24–48 h	NR	Titanium mesh
Reich et al. (2014) [33]	prospective	10	26–83	8/2	06.2011–11.2013	6 m	0/10	NR	subciliary (3), transconjunctival (4), mediopalpebral (2), transmaxillary (1)	Titanium mesh (Matrix MIDFACE, Synthes, Switzerland)
O’Connell et al. (2015) [34]	retrospective	20	29, 19–57	18/2	10 years	26 m, 2–120 m	0/20	11 d, 5–19 d	subciliary	Autogenous iliac crest bone
Shah et al. (2013) [35]	retrospective	56	NR	NR	03.2009–03.2012	6 w	16/40	12 d, 1–37 d	NR	NR
Ishida et al. (2016) [36]	retrospective	5	39.6, 19–67	3/2	03.2005–04.2016	NR	2/3	NR	NR	NR
Ploder et al. (2003) [37]	retrospective	30	45.3, 22–70	22/8	01.2000–12.2001	12 w	10/20	5.6 d, 2.1–9.1 d	endoscopic endonasal (11), endoscopic endonasal & transconjunctival (9)	Balloon catheter (11), balloon catheter & Ethisorb^®^ patch (9)
Sigron et al. (2021) [38] *	retrospective	30	51.2, 20–91	15/15	05.2016–11.2018	6 m	0/30	4.1 ± 3.1 d (13) 4.2 ± 5.2 d (17)	mid-eyelid (28), transconjunctival (1), laceration (1)	Titanium conventional (13) & preformed titanium mesh implant (17), (MatrixMIDFACE, Synthes, Switzerland or MODUS OPS 1.5, Medartis, Switzerland)
Soejima et al. (2013) [39]	retrospective	30	19–75	21/9	06.2006–11.2011	6 m	0/30	13.9 d, 10.3–17.5 d	endoscopic transmaxillary	Balloon catheter
Polligkeit et al. (2013) [40]	prospective	13	43.2, 18–82	7/6	02.2009–08.2012	NR	0/13	9.4 d, 3.6–15.2 d	combined endoscopic transmaxillary, subciliary	PDS™ sheets (7), preformed titanium implants (3), (MatrixORBITAL Synthes, Switzerland) Ethisorb^®^ patch (3)
Homer et al. (2019) [41]	retrospective	22	47.1	16/6	01.2015–04.2016	21 m	14/8	NR	NR	NR
Abdelazem et al. (2020) [42]	prospective	5	31	4/1	NR	3 m	0/5	NR	endoscopic transmaxillary	Titanium mesh implant (Stryker, USA)
Persons & Wong (2002) [43]	retrospective	5	18–41	4/1	NR	3 m–1 y	0/5	NR	endoscopic transmaxillary	LactoSorb^®^
Jin et al. (2007) [44]	retrospective	45	30	76/24%	1992–2004	3 m–4 y	0/45	0–7 (4), 8–14 (26), 15–21 (8), >22 (7)	endoscopic endonasal (1), transmaxillary (16), external (28)	Balloon catheter, Medpor^®^ implant
Ethunandan & Evans (2011) [45]	retrospective	3	21–53	2/1	NR	5.1 m	0/3	12.3 d	transcutaneous, transconjunctival	MacroPore^®^ implant
Al-Qattan & Al-Qattan (2021) [46]	retrospective	7	35, 25–50	7/0	20 y	11 m, 6–16 m	0/7	<2 d	infraorbital	Titanium mesh
Karthik et al. (2019) [47]	retrospective	3	24–29	2/1	06.2012–01.2017	12 m	0/3	<6 d	subciliary, endoscopic endonasal	NR
Sigron et al. (2020) [48] *	retrospective	22	49.8, 20–83	12/10	05.2016–11.2018	6 m	0/22	4.1 ± 3.1 d (12) 2.8 ± 2.5 d (10)	mid-eyelid, transconjunctival, transcaruncular	Freehand (12) & pre-bent patient-specific titanium mesh implant (10), (MatrixMIDFACE, Synthes, Switzerland or MODUS OPS 1.5, Medartis, Switzerland)
Emodi et al. (2018) [49]	retrospective	9	32.7, 24–48	7/2	2008–2016	1–3 y	0/9	1–4 d	combined transmaxillary and midtarsal (4), subciliary (3), infraorbital (2) + intraoral	Maxillary antral bone grafts
Cheong et al. (2010) [50]	retrospective	13	21.15, 18–38	9/7	04.1998–06.2008	27.5 m, 4 m–10 y	0/13	1–5 d (6), 8–11 d (2), 23–95 (4), 5 y (1)	endoscopic transmaxillary	Titanium micromesh (7) Medpor^®^ implant (5), NR (1)

Abbreviations: d = days, w = weeks, m = months, y = years, no = number, NR = not reported, * = Studies share the same patient population.

**Table 2:** Summary of the reported mean durations of surgical interventions for orbital floor fracture reconstruction, categorized by surgical approach and reconstruction method.

Source	Mean Duration [min]	Method of Reconstruction	Surgical Approach
Nahlieli et al. (2007) [7]	60–120	titanium mesh implant	endoscopic transmaxillary
Fernandes et al. (2007) [29]	70–80	Medpor implant	endoscopic transmaxillary
Reich et al. (2014) [33]	82	balloon catheter	transmaxillary
Reich et al. (2014) [33]	79	PDS sheets	subciliary, transconjunctival, mediopalpebral, wound
Reich et al. (2014) [33]	110	preformed titanium mesh implant	
Reich et al. (2014) [33]	76	others	combination
Sigron et al. (2020) [48]	99.8 ± 28.9	freehand bent titanium mesh implant	mid-eyelid, transconjunctival, transcaruncular
Sigron et al. (2020) [48]	57.3 ± 23.4	pre-bent patient-specific titanium mesh implant	mid-eyelid, transconjunctival, transcaruncular

**Table 3:** Overview of the injury mechanisms leading to isolated orbital floor fractures as reported in the included studies.

Source	Location	Assault [No.]	MVAs [No.]	Work [No.]	Sport [No.]	Blunt Trauma [No.]	Fall [No.]	GWs [No.]	Others [No.]
Nahlieli et al. (2007) [7]	Israel	6	0	2	1	0	0	0	0
Yano et al. (2010) [25]	Japan	0	0	0	2	0	0	0	0
Ikeda et al. (1999) [27]	Japan	2	2	0	2	0	0	0	0
Prabhu et al. (2021) [28]	USA	26	22	0	0	13	13	1	0
Fernandes et al. (2007) [29]	USA	6	4	0	0	0	0	0	0
Gugliotta et al. (2023) [31]	Italy	8	0	0	9	0	1	0	0
Worthington (2010) [32]	New Zealand	0	0	0	5	0	0	0	0
Reich et al. (2014) [33]	Germany	1	0	0	1	0	7	0	1
O’Connell et al. (2015) [34]	Ireland	8	0	0	11	0	1	0	0
Ishida et al. (2016) [36]	Japan	1	0	0	1	0	3	0	0
Ploder et al. (2003) [37]	Austria	12	3	0	5	0	8	0	2
Sigron et al. (2021) [38]	Switzerland	9	1	1	3	0	16	0	0
Soejima et al. (2013) [39]	Japan	12	0	0	9	0	9	0	0
Abdelazem et al. (2020) [42]	Egypt	0	3	0	1	1	0	0	0
Ethunandan & Evans (2011) [45]	United Kingdom	2	0	0	1	0	0	0	0
Al-Qattan & Al-Qattan (2021) [46]	Saudi Arabia	0	0	0	0	7	0	0	0
Karthik et al. (2019) [47]	India	1	2	0	0	0	0	0	0
		**Assault**	**MVAs**	**Work**	**Sport**	**Blunt trauma**	**Fall**	**GWs**	**Others**
**TOTAL [No.]**	**268**	**94**	**37**	**3**	**51**	**21**	**58**	**1**	**3**
**TOTAL [%]**		**35.1**	**13.8**	**1.1**	**19.0**	**7.9**	**21.6**	**0.4**	**1.1**

Abbreviations: MVAs = motor vehicle accidents, GWs = gunshot wounds, No. = number.

**Table 4:** Summary of the presence of enophthalmos greater than 2 mm before and after surgery in 262 patients. The table does not differentiate between conservative and surgical management in all cases.

Source	Patients [No.]	Preoperative Enophthalmos [No.]	Postoperative Enophthalmos [No.]
Nahlieli et al. (2007) [7]	5	3	0
Scolozzi et al. (2009) [26]	7	2	0
Ikeda et al. (1999) [27]	6	1	0
Prabhu et al. (2021) [28]	75	36	7
Scawn et al. (2016) [30]	10	7	0
O’Connell et al. (2015) [34]	20	2	1
Ishida et al. (2016) [36]	5	0	0
Sigron et al. (2021) [38]	30	6	1
Soejima et al. (2013) [39]	30	4	1
Polligkeit et al. (2013) [40]	13	2	0
Jin et al. (2007) [44]	17 (endoscopic)	4	0
Jin et al. (2007) [44]	28 (external)	10	3
Karthik et al. (2019) [47]	3	3	0
Cheong et al. (2010) [50]	13	8	0
**TOTAL [No.]**	**262**	**88**	**13**
**TOTAL [%]**		**33.6**	**5.0**
**Persistence rate after surgery [%]**			**14.8**
**Resolution rate after surgery [%]**			**85.2**

Abbreviations: No. = number.

**Table 5:** Comparison of preoperative and postoperative diplopia symptoms in 398 patients. The table does not differentiate between conservative and surgical management in all cases.

Source	Patients [No.]	Preoperative Diplopia [No.]	Postoperative Diplopia [No.]
Nahlieli et al. (2007) [7]	5	4	0
Yano et al. (2010) [25]	2	2	0
Scolozzi et al. (2009) [26]	7	5	1
Prabhu et al. (2021) [28]	75	38	13
Fernandes et al. (2007) [29]	10	9	0
Scawn et al. (2016) [30]	10	5	1
Worthington (2010) [32]	5	5	0
Reich et al. (2014) [33]	10	10	3
O’Connell et al. (2015) [34]	20	19	3
Shah et al. (2013) [35]	56	19	4
Ishida et al. (2016) [36]	5	3	0
Ploder et al. (2003) [37]	30	18	1
Sigron et al. (2021) [38]	30	19	8
Soejima et al. (2013) [39]	30	30	2
Polligkeit et al. (2013) [40]	13	8	6
Abdelazem et al. (2020) [42]	5	3	0
Persons & Wong (2002) [43]	5	3	0
Jin et al. (2007) [44]	17 (endoscopic)	15	7
Jin et al. (2007) [44]	28 (external)	22	13
Ethunandan & Evans (2011) [45]	3	3	1
Al-Qattan & Al-Qattan (2021) [46]	7	7	0
Karthik et al. (2019) [47]	3	3	1
Emodi et al. (2018) [49]	9	5	0
Cheong et al. (2010) [50]	13	7	2
**TOTAL [No.]**	**398**	**262**	**66**
**TOTAL [%]**		**65.8**	**16.6**
**Persistence rate after surgery [%]**			**25.2**
**Resolution rate after surgery [%]**			**74.8**

Abbreviations: No. = number.

**Table 6:** Summary of the preoperative and postoperative infraorbital hypoesthesia in 78 patients. The table does not differentiate between conservative and surgical management in all cases.

Source	Patients [No.]	Preoperative Hypoesthesia [No.]	Postoperative Hypoesthesia [No.]
Nahlieli et al. (2007) [7]	5	4	2
Yano et al. (2010) [25]	2	1	2
Ikeda et al. (1999) [27]	6	2	0
Scawn et al. (2016) [30]	10	5	1
Sigron et al. (2021) [38]	30	14	7
Homer et al. (2019) [41]	22	7	2
Ethunandan & Evans (2011) [45]	3	3	0
**TOTAL [No.]**	**78**	**36**	**14**
**TOTAL [%]**		**46.2**	**17.9**
**Persistence rate after surgery [%]**			**38.9**
**Resolution rate after surgery [%]**			**61.1**

Abbreviations: No. = number.

**Table 7:** Summary of the pre- and postoperative ocular motility restriction in 156 patients.

Source	Patients [No.]	Preoperative Motility Restriction [No.]	Postoperative Motility Restriction [No.]
Nahlieli et al. (2007) [7]	5	2	0
Yano et al. (2010) [25]	2	2	0
Ikeda et al. (1999) [27]	11	2	0
Prabhu et al. (2021) [28]	75	45	23
Scawn et al. (2016) [30]	10	6	2
Sigron et al. (2021) [38]	30	14	4
Polligkeit et al. (2013) [40]	13	7	4
Abdelazem et al. (2020) [42]	5	3	0
Ethunandan & Evans (2011) [45]	3	3	0
Karthik et al. (2019) [47]	2	2	0
**TOTAL [No.]**	**156**	**86**	**33**
**TOTAL [%]**		**55.1**	**21.2**
**Persistence rate after surgery [%]**			**38.4**
**Resolution rate after surgery [%]**			**61.6**

Abbreviations: No. = number.

## Data Availability

The data presented in this study are available on request from the corresponding author due to possible licensing restrictions related to the original publications.

## Abbreviations

The following abbreviations are used in this manuscript:

CT	computed tomography
d	days
GWs	gunshot wounds
JBI	the Joanna Briggs Institute
m	months
MRI	magnetic resonance imaging
MVAs	motor vehicle accidents
No	number
NR	not reported
PDS	polydioxanone sheets
PRISMA	Preferred Reporting Items for Systematic Reviews and Meta-Analyses
w	weeks
y	years

## Appendix A

**Table A1:** Overview of the quality assessment results for all included cohort studies, based on the Joanna Briggs Institute (JBI) Checklist for Cohort Studies [22].

Source	1	2	3	4	5	6	7	8	9	10	11
Nahlieli et al. (2007) [7]											
Yano et al. (2010) [25]											
Scolozzi et al. (2009) [26]											
Ikeda et al. (1999) [27]											
Prabhu et al. (2021) [28]											
Fernandes et al. (2007) [29]											
Scawn et al. (2016) [30]											
Gugliotta et al. (2023) [31]											
Worthington (2010) [32]											
Reich et al. (2014) [33]											
O’Connell et al. (2015) [34]											
Shah et al. (2013) [35]											
Ishida et al. (2016) [36]											
Ploder et al. (2003) [37]											
Sigron et al. (2021) [38] ***											
Soejima et al. (2013) [39]											
Polligkeit et al. (2013) [40]											
Homer et al. (2019) [41]											
Abdelazem et al. (2020) [42]											
Persons & Wong (2002) [43]											
Jin et al. (2007) [44]											
Ethunandan & Evans (2011) [45]											
Al-Qattan & Al-Qattan (2021) [46]											
Karthik et al. (2019) [47]											
Sigron et al. (2020) [48] ***											
Emodi et al. (2018) [49]											
Cheong et al. (2010) [50]											

Color Coding: green = yes, red = no, orange = unclear, grey = not applicable. * = studies share the same patient population.
